# Causes of the Failure of Biological Therapy at a Tertiary Center: A Cross-Sectional Retrospective Study

**DOI:** 10.7759/cureus.18253

**Published:** 2021-09-24

**Authors:** Arwa Z Fatani, Nada A Bugshan, Hanan M AlSayyad, Mayar A Shafei, Nada M Hariri, Laila T Alrashid, Ahlam Y Lasker, Badreyah A Aldauig, Suzan M Attar

**Affiliations:** 1 Faculty of Medicine, King Abdulaziz University, Jeddah, SAU; 2 Department of Internal Medicine, King Abdulaziz University, Jeddah, SAU

**Keywords:** rheumatoid arthritis, biological therapy, refractory disease, failures, tnf inhibitors, rituximab

## Abstract

Introduction

Rheumatoid arthritis (RA) is one of the most commonly encountered autoimmune diseases. Treatment generally includes disease-modifying anti-rheumatic drugs (DMARDs) and/or biological therapy. However, a significant proportion of the patients do not respond to treatment either as a (primary failure) or lose efficacy over time (secondary failure). Several factors are assumed to influence these conditions.

Objectives

To estimate the prevalence of failure of biological therapy in patients with RA and its causes.

Methods

A total of 335 RA patients who were diagnosed at a tertiary center in Jeddah, Saudi Arabia, and had a failure after receiving biological therapy were included in this study. Several variables were considered; patient’s socio-demographic data, comorbid conditions, types of biological therapy, the duration of using biological therapy in months, number of biological therapies, allergic reactions, disease activity, and treatment duration.

Results

Overall the prevalence of failure to biological therapy was 58%; 77% primary failure and 23% secondary failure. Patients with negative rheumatoid factor (RF) (p=0.006), using low-dose steroids, and with a longer disease duration had a significant failure of biological therapy (p=0.023).

Conclusion

A high percentage of RA patients had a failure of biological therapy. A multicentric trial is recommended to look for additional factors.

## Introduction

The principal rheumatoid arthritis (RA) treatments are disease-modifying anti-rheumatic drugs (DMARDs) and/or biological DMARDs (bDMARDs) [1]. The introduction of bDMARDs designed to inhibit particular cellular or molecular targets specifically involved in the pathogenesis of the disease has significantly enhanced the outcome of RA therapy, resulting in clinical recovery or decreased activity of the disease. However, patients may also discontinue or delay care due to unfavored side effects. A randomized controlled trial in Greece showed that a considerable number of patients discontinued anti-tumor necrosis factor (TNF) therapy in 2019 either due to primary failure, secondary lack of response, or intolerance. Primary failure was observed in a considerable percentage of RA patients who don't respond to bDMARDs. Treatments that are possibly a result of non-TNF inflammatory pathways may be dominant in individual patients. In a previous study, it was found that starting DMARDs at a younger age, a high baseline disease activity score (DAS)-28 score, poorer early response within the first six months of treatment with bDMARDs (estimated by delta-DAS-28), and the presence of erosions were correlated with multi-refractoriness. However, anti-TNF agents are not effective in all patients. About 30% of patients treated with a TNF inhibitor failed to achieve an improvement of 20% in American College of Rheumatology criteria (ACR20; primary failure or inefficacy), and more patients lose efficacy during therapy (secondary failure or acquired therapeutic resistance) or experience adverse events following treatment with a TNF inhibitor. In addition, others may demonstrate primary response initially and then develop over time (secondary failure) as a decline of efficacy. There is a previous study that documented the frequency of patients who do not achieve even the weakest response to the standard dosage of anti-TNF agents, which ranges between 28% and 58%, namely, ACR20 (20% improvement) response. Efficacy and adverse effects were the major factors for the discontinuation. The other factors are genetic mutations such as FAS-L and Caspase-9 in the apoptosis-related genes. There are other contributing factors to secondary failure of bDMARDs, such as a longer period of illness lasting more than two years, smoking, and small bowel involvement [2]. An additional cause that influences TNF-α inhibitors' secondary failure is anti-drug antibodies development [3]. Methotrexate (MTX) discontinuation after the initiation of bDMARDs, adherence to treatment, or variations in the pharmacokinetics of TNF-α inhibitors. Still, there aren't enough studies in the literature. Therefore, we aimed to determine retrospectively the causes of bDMARDs failure in RA patients in a tertiary center.

## Materials and methods

A retrospective study was conducted from April 2015 to January 2019 at a tertiary center. The ethical committee approved this study. Our sample size comprised 335 patients (51 (11.9%) of male patients) and (284 (66.2%) of female) patients who had either primary or secondary failure to RA treatment. Primary failure is generally defined as no clinical response within the initial treatment while secondary failure is defined as loss of effectiveness of the drug after initial remission. The data included admitted patients and those in the database. Those who failed to respond to bDMARDs were not able to progress to a better prognosis and the desired treatment plan. While patients experiencing infection, pregnancy, or had recent cancer <5 years were excluded. The medical records were obtained, collected, and evaluated using a dedicated data extraction sheet along with examining the patient in the clinic. These records consisted of the patient’s sociodemographic data based on their age, gender, nationality, body mass index (BMI), weight, height, smoking, disease duration, family history of RA, and a history of abortions and pregnancies. The comorbid conditions were diabetes mellitus, tuberculosis (TB), hypertension, liver cirrhosis, hyperlipidemia, cardiovascular diseases, cancer, lymphoma, chronic obstructive pulmonary disease (COPD), asthma. The medications were methotrexate, steroids, aspirin, non-steroidal anti-inflammatory drugs (NSAIDs), and traditional DMARDs. The use of bDMARDs included adalimumab, etanercept, infliximab, rituximab, and tocilizumab. The records also included the duration of using bDMARDs in months, the name of the first bDMARDs, the number of current bDMARDs, and the name of the second bDMARDs. Also, the study measured local and general allergic reactions and the lab results were rheumatoid factor (RF) positive or negative, RF level, anti-cyclic citrullinated peptide (CCP) level, erythrocyte sedimentation rate (ESR) level, c-reactive protein (CRP) level, and antinuclear antibodies (ANA) level. Disease activity was measured by the DAS-28 score and the result was measured as well. Data entry was done using Microsoft Excel 2016 (Microsoft Corporation, Redmond, WA) and statistical analysis was performed by using the Statistical Package for the Social Sciences (SPSS) software, version 21 (IBM Corp., Armonk, NY). Categorical variables, including primary variables, were described using frequencies. Continuous variables for normally distributed were described using means and standards. A univariate analysis was conducted for categorical variables using the chi-square test to check for all the possible causes. A test with a p-value of <0.05 was considered significant. All information in this study was confidential, with no access to data other than to those authorized.

## Results

Twenty-six percent (26%) of studied patients had an age ranging from (40 to <50 years), 84.8% were females with 2.5% who had a previous pregnancy and 7.4% who had a previous abortion. Of these patients,65.1% were of Saudi nationality, 15.2% were current smokers, and 32.2% were obese. Most of the patients (89.6%) were on monotherapy biological treatment, 19.1% had a family history of RA, and 43% had RF (Table 1).

**Table 1:** Distribution of studied patients according to their characters, smoking, BMI categories, currently used biological therapy, and a family history of RF Ex-smoker; no longer smoking, RF; rheumatoid factor, RA; rheumatoid arthritis

Variable		No. (%)
Age	<40	98 (9.3)
	40 - <50	87 (26)
	50 - <60	762 (22.7)
	≥60	74 (22.1)
Gender	Male	51 (15.2)
	Female	284 (84.8)
For females	Pregnancy	
	Yes	7 (2.5)
	No	277 (97.5)
	Previous abortion	
	Yes	21 (7.4)
	No	263 (92.6)
Nationality	Saudi	218 (65.1)
	Non- Saudi	117 (34.9)
Smoking	Yes	51 (15.2)
	No	274 (81.8)
	Ex-smoker	10 (3)
BMI categories	Underweight	6 (1.8)
	Normal	56 (16.7)
	Over	74 (22.1)
	Obesity	108 (32.2)
	Severe obesity	91 (27.2)
Current biological therapy	Monotherapy	300 (89.6)
	Two types	21 (6.3)
	Three types	14 (4.2)
Family history of RA	Yes	64 (19.1)
	No	271 (80.9)
RF	Yes	144 (43)
	No	182 (54.3)
	Not applicable	9 (2.7)

Table 2 shows that 4.8% of patients had infertility, 12.2% had TB and most of it was of the pulmonary type (67.4%). More than half of the patients (59.4%) had comorbidities, where the most common was vasculitis (39.4%), hypertension (HTN; 29%), diabetes mellitus (DM; 28.1%), and hyperlipidemia (23.9%).

**Table 2:** Distribution of studied patients according to the presence of infertility, TB, and comorbidities TB; tuberculosis, DM; diabetes mellitus, HTN; hypertension, CVD; cardiovascular diseases, COPD; chronic obstructive lung disease, SLE; systemic lupus erythematous

Variable		No. (%)
Infertility	Yes	16 (4.8)
	No	319 (95.2)
TB	Yes	41 (12.2)
	No	294 (87.8)
Old TB	Yes	30 (9)
	No	305 (91)
Latent TB	Yes	20 (6)
	No	315 (94)
TB type	Bone	5 (11.6)
	Abdomen	5 (11.6)
	Potts	4 (9.3)
	Pulmonary	29 (67.4)
Co-morbidity	Yes	199 (59.4)
	No	136 (40.6)
Co-morbid disease	DM	94 (28.1)
	HTN	97 (29)
	CVD	31 (9.3)
	Hyperlipidemia	80 (23.9)
	Hypoalbuminemia	47 (14)
	Renal impairment	46 (13.7)
	Previous lung diseases	64 (19.1)
	Asthma	29 (8.7)
	COPD	18 (5.4)
	Lymphoma	19 (5.7)
	Vasculitis	132 (39.4)
	Osteoporosis	40 (11.9)
	SLE	41 (12.2)
	Psoriasis	30 (9)
	Cirrhosis	1 (0.3)
	Fatty liver	58 (17.3)
	Cancer:	34 (10.1)
	Cancer thyroid	1 (2.9)
	Cancer lung	13 (38.2)
	Multiplemyloma	7 (20.6)
	Cancer breast	4 (11.8)
	Cancer bladder	4 (11.8)
	Cancer colon	5 (14.7)

Table 3 demonstrated that 9.3% of patients had an allergic reaction to taken biological therapy and the most common allergic drug was rituximab (41.9%). Of the patients, 49.6% had anti-CCP, 57.6% had anti-mutated citrullinated vimentin (MCV), 51.3% had high ESR, 70.4% had a CRP level >3, and 31.4% had a double-stranded DNA (dsDNA) level of 0-200. Most of the patients (60%) had a positive ANA, 83.6% had disease activity, and 48.7% had a DAS-28 of 3.2-5.1, 46.6% had an aspartate aminotransferase (AST) of 10-40, 49% had an alanine transaminase (ALT) of 7-56, and 19.4% had an acetylsalicylic acid (ASA) of 65. Most of the patients were using NSAIDs (80.6%), 56.7% were using a steroid dose of <5 mg, 80% were using Methtrx, and 78.5% were using Methotrexate (MTX). The mean disease duration (months), HB, platelet, and creatine levels, and MTX dose were 38.91 ± 70.16 months, 11.78 ± 1.73, 324.24 ± 100.71, 88.65 ± 389.67, and 8.68± 4.41, respectively.

**Table 3:** Distribution of studied patients according to the allergic reaction to taken biological therapy, clinical and laboratory data, and drugs used Anti-CCP; anti-cyclic citrullinated peptide, Anti-MCV; anti-mutated citrullinated vimentin, ESR; erythrocyte sedimentation rate, CRP; C-reactive protein, dsDNA; double-stranded DNA, ANA; antinuclear antibodies, DAS-28; disease activity score, AST; aspartate aminotransferase, ALT; alanine transaminase, NSAIDs; nonsteroidal anti-inflammatory drugs, HB level; hemoglobin level, MTX dose; methotrexate dose, ASA; acetylsalicylic acid

Variable		No. (%)
Allergic reaction to taken biological therapy	Yes	31 (9.3)
	No	304 (90.7)
If yes, what is the allergic drug?	Rituximab	13 (41.9)
	Infliximab	6 (19.4)
	Adalimumab (Humira)	4 (12.9)
	Etanercept (Enbrel)	4 (12.9)
	Tocilizumab	4 (12.9)
Anti-CCP	Yes	166 (49.6)
	No	169 (50.4)
Anti-MCV	Yes	193 (57.6)
	No	141 (42.1)
	Not applicable	1 (0.3)
ESR	High	172 (51.3)
	Normal	163 (48.7)
CRP	>3	236 (70.4)
	≤ 3	98 (29.3)
	Not applicable	1 (0.3)
dsDNA	0-200	41.5 (31.4)
	201-300	30.1 (30.1)
	301-800	26.9 (26.9)
	>800	1.5 (1.5)
ANA	Positive	201 (60)
	Negative	134 (40)
Activity	Yes	280 (83.6)
	No	55 (16.4)
DAS-28	≤ 2.6	2 (0.6)
	2.6-3.2	114 (34)
	3.2-5.1	163 (48.7)
	≥ 5.1	56 (16.7)
	≤ 2.6	2 (0.6)
AST	<10	93 (27.8)
	10-40	156 (46.6)
	>40	86 (25.7)
ALT	<7	93(27.8)
	7-56	164 (49)
	>56	78 (23.3)
ASA	Yes	65 (19.4)
	No	270 (80.6)
NSAIDs	Yes	270 (80.6)
	No	65 (19.4)
Steroid dose	<5mg	190 (56.7)
	5-10mg	133 (39.7)
	>10mg	12 (3.6)
Used drugs	Methotrexate	263 (78.5)
	Antimalarial	107 (31.9)
	Methtrx	268 (80)
	Sulfa	60 (17.9)
	Avara	49 (14.6)
	Immurane	47 (14)
Mean and SD of study variables	Disease duration (months)	38.91 ± 70.16
	HB level	11.78 ± 1.73
	Platelet level	324.24 ± 100.71
	Creatine level	88.65 ± 389.67
	MTX dose	8.68± 4.41

Of the studied patients, 58.5% failed to respond to biological therapy. In contrast, only 41.5% responded to biological therapy. 

**Figure 1:**
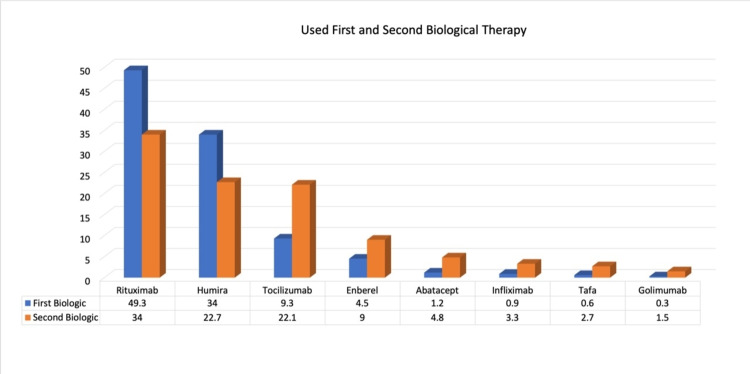
Percentage distribution of patients according to used first and second biological therapy

Figure 1 shows that most of the patients were using Humira (34%) and rituximab (49.3%) as the first biological therapy and Humira (22.7%) and rituximab (34%) as the second biological therapy.

Table 4 shows that patients of Saudi nationality and those who did not have RF had a significantly higher percentage of those who had a failure of biological therapy (p=< 0.05). On the other hand, a non-significant difference was found between failure of biological therapy and other patient characteristics such as smoking, BMI categories, and currently used biological therapy.

**Table 4:** Relation to failure of biological therapy and patients’ characteristics, smoking, BMI categories, currently used biological therapy, and family history of RF RA; rheumatoid arthritis, RF; rheumatoid factor, BMI; body mass index

Variable		Biological failure		χ2	P-value
		Present No. (%)	Absent No. (%)		
Age	<40	37 (37.8)	61 (62.2)	1.75	0.627
	40 - <50	41 (47.1)	46 (52.9)		
	50 - <60	31 (40.8)	45 (59.2)		
	≥ 60	30 (40.5)	44 (59.5)		
Gender	Male	22 (43.1)	29 (56.9)	0.06	10.796
	Female	117 (41.2)	167 (58.8)		
Nationality	Saudi	80 (36.7)	38 (63.3)	5.91	0.015
	Non- Saudi	59 (50.4)	58 (49.6)		
Smoking	Yes	25 (49)	26 (51)	3.08	0.214
	No	108 (39.4)	166 (60.6)		
	Ex-smoker	6 (60)	4 (40)		
BMI categories	Underweight	0 (0.0)	6 (100)	5.14	0.273
	Normal	22 (39.3)	34 (60.7)		
	Over	29 (3.2)	45 (60.8)		
	Obesity	48 (44.4)	60 (55.6)		
	Severe obesity	40 (44)	51 956)		
Family history of RA	Yes	32 (50)	32 (50)	2.35	0.125
	No	107 (39.5)	164 (60.5)		
RF	Yes	74 (51.4)	70 (48.6)	10.18	0.001
	No	65 (34)	126 (66)		

Table 5 shows that patients with vasculitis had a significantly higher percent of those who had a failure of biological therapy (p=< 0.05). On the other hand, a non-significant difference was found between the failure of biological therapy and infertility, TB, and other comorbidities (cancer (χ2=1.3, p-value=0,254), COPD (χ2=1.55, p-value=0.213), asthma (χ2=3.83, p-value=0.05), previous lung diseases (χ2=0.47, p-value=0.47), renal impairment (χ2=0.38, p-value=0.538), hypoalbuminemia (χ2=0.63, p-value=0.424), hyperlipidemia (χ2=0.22, p-value=0.639), CVD (χ2=2.5, p-value=0.113), DM (χ2=0.54, p-value=0.459), osteoporosis (χ2=0.67, p-value=0.411), SLE (χ2=0.46, p-value=496), and psoriasis (χ2=0.04, p-value=0.63)) (p=>0.05).

**Table 5:** Relation to failure of biological therapy and presence of infertility, TB, and comorbidities TB; tuberculosis

Variable		Biological failure		χ2	P-Value
		Absent No. (%)	Present No. (%)		
TB	Yes	23 (56.1)	18 (43.9)	0.11	0.73
	No	173 (58.8)	121 (41.2)		
Comorbidity	Yes	116 (58.3)	83 (41.7)	0.009	0.92
	No	80 (58.8)	56 (41.2)		

Table 6 shows that patients who had an allergic reaction to taken biological therapy, those who used NSAIDs and who had a steroid dose <5 mg, who were not using antimalarial drugs, sulfa, or Immurane, and those with a longer disease duration had a significantly higher percentage of those who had a failure of biological therapy (p=<0.05). On the other hand, a non-significant difference was found between the failure of biological therapy and other clinical and laboratory data and other drugs used (Methotrexate (DMARD) (χ2=0.05, p-value=0.813), Methtrx (MTX) (χ2=0.37, p-value=0.542), and Avara (χ2=2.14, p-value=0.143)) (p=>0.05).

**Table 6:** Relation of failure of biological therapy and allergic reaction to taken biological therapy, clinical and laboratory data, and drugs used * Mann-Whitney test Anti-CCP; Anti-cyclic citrullinated peptide, Anti-MCV; Anti-mutated citrullinated vimentin, ESR; erythrocyte sedimentation rate, CRP; C-reactive protein, dsDNA; double-stranded DNA, ANA; antinuclear antibodies, DAS-28; disease activity score, NSAIDs; nonsteroidal anti-inflammatory drugs, HB level; hemoglobin level, MTX; methotrexate

Variable		P-value		χ2	P-value
		Absent No. (%)	Present No. (%)		
Allergic reaction to taken biological therapy	Yes	31 (100)	0 (0.0)	24.22	< 0.001
	No	165 (54.3)	139 (45.7)		
Anti-CCP	Yes	96 (57.8)	70 (42.2)	0.06	0.803
	No	100 (59.2)	69 (40.8)		
Anti-MCV	Yes	11 (57.5)	82 (42.5)	1.67	0.433
	No	85 (60.3)	56 (39.7)		
	Not applicable	0 (0.0)	1 (100)		
ESR	High	97 (56.4)	75 (43.6)	0.65	0.42
	Normal	99 (60.7)	64 (39.3)		
CRP	> 3	135 (57.2)	101 (42.8)	2.13	0.343
	≤ 3	61 (82.2)	37 (37.8)		
	Not applicable	0 (0.0)	1 (100)		
dsDNA	0-200	75 (45)	64 (46)	2.1	0.53
	201-300	61 (60.4)	40 (39.6)		
	301-800	57 (63.3)	33 (36.7)		
	> 800	3 (60)	2 (40)		
ANA	Positive	114 (56.7)	87 (43.3)	0.66	0.415
	Negative	82 (61.2)	52 (88.8)		
Activity	Yes	164 (58.6)	116 (41.4)	0.003	0.957
	No	32 (58.2)	23 (41.8)		
DAS-28	≤ 2.6	2 (100)	0 (0.0)	5.35	0.148
	2.6-3.2	70 (61.4)	441 (38.6)		
	3.2-5.1	98 (60.1)	65 (39.9)		
	≥ 5.1	26 (46.4)	30 (53.6)		
NSAIDs	Yes	169 (62.6)	101 (37.4)	9.56	0.002
	No	27 (41.5)	38 (58.5)		
Steroid dose	< 5mg	129 (67.9)	61 (32.1)	20.88	< 0.001
	5-10mg	64 (48.1)	69 (51.9)		
	> 10mg	2 (18.2)	9 (81.8)		
	Not applicable	1 (100)	0 (0.0)		
Quantitative variables	Duration	47.55± 85.27	26.55± 36.48	3.74*	< 0.001
	HB level	11.73 ± 1.72	11.85± 1.75	0.85*	0.393
	Platelet level	330.59±114.47	315.29 ±76.7	0.87*	0.38
	Creatine level	96.93± 50.8	76.98± 75.93	1.04*	0.298
	MTX Dose	8.58 ± 4.58	8.82 ± 4.18	0.81*	0.215

By binary logistic regression analysis, using a steroid dose <5 mg was an independent predictor (risk factor) for the failure of biological therapy among studied patients (p=<0.05) as shown in Table 7.

**Table 7:** Binary logistic regression analysis of risk factors of failure to biological therapy NSAIDs; nonsteroidal anti-inflammatory drugs, RF; rheumatoid factor

Variable	P-value	Odds ratio
Nationality	0.143	1.48
RF	0.64	0.65
Vasculitis	4.66	1.23
Allergic reaction to taken biological therapy	0.998	5.04
NSAID	0.165	1.58
Steroid dose	0.023	1.79
Duration	0.177	0.99

## Discussion

This study estimated that more than half of RA patients had primary failure compared to secondary failure of biological therapy (bDMARD). More than half of the patients had comorbid conditions similar to previously conducted studies [4-6]. Patients who were RF negative had a significantly higher percentage of failure of biological therapy, contrary to a study that included 400 patients receiving bDMARD in the city of Bogotá, Colombia, which demonstrated that RF-negative patients have frequent remission and lower levels of disability compared to RF-positive patients treated with anti-TNF alpha agents [7]. Furthermore, an observational study was done between January 2000 and August 2019, which reported that the later that bDMARD was initiated and longer disease duration are prone to have multi-refractory diseases, as they present with advanced disease courses. Thus, early intervention with biological therapy is recommended in order to establish beneficial treatment outcomes [5]. Our data demonstrated that the higher the dose of steroids, the better the outcome, as most of our patients who received >10 mg/d had less failure of biological therapy (18.2%). Marije F. Bakker et al. showed that the inclusion of prednisone 10 mg/d from the start of an MTX-based, tight-control strategy slows erosive joint damage and further enhances clinical efficacy [8]. A non-significant relation was found between the failure of biological therapy and smoking compared to multiple studies that proved that smoking reduced the effect of both non-biological and biological DMARDs in RA treatment [7-9]. Most of our sample were females, as they are more likely to attain autoimmune disorders due to the hormonal changes women experience [10].

To date, no research has studied the prevalence of failure of biological therapy in the Middle East. Nevertheless, further work is valuable to identify the genetic and other constitutional factors, which may be linked to the disease activity that may determine the response of the failure to biological treatment, which is improbable to be a random effect. This study has several limitations, including the fact that it has a single-center, hospital-based, cross-sectional design. However, this is one of few studies that assessed the disease activity by accurate clinical measures using DAS-28. Larger, prospective, multi-centric cohort studies are needed to highlight the advocacy of applying DAS-28 by physicians, consider failure as a complication in all RA patients, and be updated on the next step of management if so.

## Conclusions

A high percentage of RA patients had a failure of bDMARDs. A multicentric trial is recommended to look for additional patient-related, drug, genetic, and environmental factors.
